# Immunotherapy for Peritoneal Metastases from Gastric Cancer: Rationale, Current Practice and Ongoing Trials

**DOI:** 10.3390/jcm10204649

**Published:** 2021-10-11

**Authors:** Eva Ruiz Hispán, Manuel Pedregal, Ion Cristobal, Jesús García-Foncillas, Cristina Caramés

**Affiliations:** 1 Department of Oncology, Fundación Jiménez Díaz University Hospital, IIS-Fundación Jiménez Díaz, 28040 Madrid, Spain; eva.ruizh@quironsalud.es (E.R.H.); manuel.pedregal@quironsalud.es (M.P.); 2 Cancer Unit for Research on Novel Therapeutic Targets, Oncohealth Institute, IIS-Fundación Jiménez Díaz-UAM Madrid, 28040 Madrid, Spain; ion.cristobal@quironsalud.es

**Keywords:** gastric cancer, peritoneal metastasis, cytoreductive surgery, intraperitoneal chemotherapy, research, immunotherapy, cell therapy

## Abstract

Peritoneal metastases from gastric cancer play a key role in the fatal prognosis of the disease. The lack of efficacy of actual therapeutic approaches together with the outcomes achieved with checkpoint inhibitors in gastric cancer compel us to address the current state-of-the-art immunotherapy treatment of peritoneal dissemination. The immunogenicity of the peritoneum has been described to be particularly active at omentum and peritoneal lymph nodes. Also, both innate and acquired immunity seems to be involved at different molecular levels. Recent works show PDL1 expression being less present at the peritoneal level; however, some clinical trials have begun to yield results. For example, the ATTRACTION-2 trial has demonstrated the activity of Nivolumab in heavily pretreated patients even though peritoneal metastases were diagnosed in a 30% of them. Despite positive results in the metastatic setting, peritoneal responses to systemic checkpoint inhibitors remains unclear, therefore, new strategies for intraperitoneal immunotherapy are being proposed for different ongoing clinical trials.

## 1. Rationale

Gastric cancer (GC) is the fifth most diagnosed cancer and the third leading cause of cancer deaths worldwide, according to GLOBOCAN 2020 data [1]. The disease is often not found until it is at an advanced stage. Despite the use of multiple modalities to treat GC, including gastrectomy combined with radiation therapy, chemotherapy or targeted chemo-immune therapy, the disease often progresses, relapses, or metastasizes and has a five-year survival rate of less than 35% overall [2,3].

Peritoneal metastasis (PM), which is the most common form of recurrence in gastric cancer, is estimated to occur in 55–60% of gastric cancer patients.

PM [2] has only a 2% five-year overall survival rate, and this includes patients with only microscopic free cancer cells without macroscopic peritoneal nodules. The mechanism of peritoneal metastasis has yet to be fully understood, there are limited treatment options for these patients, and the appropriate target has not been identified. A complete cure through surgery is difficult and in most cases adds morbidity and mortality without impacting on increased survival, therefore, palliative systemic therapy is the first choice for treatment [4]. However, chemotherapy or other GC approved systemic therapy is often inadequate for peritoneal dissemination due to insufficient drug delivery, and to symptoms such as intestinal obstruction and abdominal bloating.

To overcome the limitations of systemic chemotherapy, a novel multimodal treatment could radically change the outcomes. This new approach combines systemic chemotherapy, radical surgery and intra-peritoneal chemotherapy (IPC) in selected patients with a peritoneal carcinomatosis index (PCI) below 12 and the possibility of a complete cytoreduction [5,6,7,8].

Novel technologies like RNA sequencing and cytometry have become increasingly important techniques to address the challenges presented by peritoneal metastases in order to understand genomics and carcinogenesis [9,10,11,12]. The development of peritoneal carcinomatosis is a multi-step process, beginning with the detachment of cancer cells from the primary tumor, followed by their attachment to peritoneal mesothelial cells, retraction of the mesothelial cells, exposure of the basement membrane, proliferation and finally growth with induction of angiogenesis [13]. In general terms, the human peritoneum is highly complex and unmatched in mice with the same tissue biology and structure. Therefore, reliable preclinical models are crucial for research and development of efficacious treatments. In this sense, syngeneic, humanized, personalized patient-derived xenograft, genetically engineered mouse models, or approaches using biotechnology for 3D tumors have offered proof of concept, enabling the preclinical study of promising immunotherapies for peritoneal carcinomatosis [14].

The immune system, between the innate (neutrophils, macrophages, dendritic cells and natural killers) and the adaptive (B and T lymphocytes) has the ability to detect and eliminate these tumor cells, which is known as immune surveillance. However, cancer cells either inherently or causatively develop strategies to escape immune surveillance by targeting or hijacking the immune system to assist their abnormal growth by a tumor microenvironment (TME) in which cancer and stromal cells participate. Since immune cells such as macrophages and lymphocytes are present in the greater omentum and lymph nodes, the activation of immune cells would be a promising strategy for treatment of PM [15].

In this sense, stimulation of the innate immune system has been described as an effective way to activate immune cells for the treatment of peritoneal dissemination. It can be carried out through dendritic cells. Since they are antigen presenting cells, they could be used as therapeutic vaccines in a co-culture with autologous T lymphocytes to educate and stimulate specific antitumor lymphocytes [16]. Also, macrophages, by the ligand recognition of the Toll-like receptor in antigen-presenting cells, stimulate Th-1-type immune responses [17] or gene therapy with the intercellular adhesion molecule of the adenovirus vector vehicle (ICAM-2) that produces NK infiltration in peritoneal metastatic lesions [18]. Immunosuppressive cell blocking strategies are also being developed, such as Mφ macrophages that are associated with the expression of PD-L1 in gastric adenocarcinoma cells [19] or Treg, with intraperitoneal arsenic trioxide (As2O3) [20].

Specific CAR-T cells are genetically engineered from patient T cells and can secrete cytokines, produce specific molecules, and exert potent cytotoxicity against a wide range of cancer cells. This strategy is being developed in the GC, with T cells modified with the chNKG2D receptor (for GC that expresses NKG2DL with peritoneal metastases) [21] and chA214-1BBz [22], in addition to the third generation bi-specific CAR-T Trop2/PD-L1 method [23].

We know that patients with GC best selected for immuno checkpoint are those with a predictive biomarker such as tumor mutational burden-high, expression of PDL-1, microsatellite instability(MSI) or Epstein-Barr virus (EBV) positive tumors, but PM shows PDL1 expression less frequently [24,25]. These findings suggest that the immune checkpoint molecules PD-1 and its ligand are unlikely treatments among possible therapeutic targets in peritoneal metastasis of GC. However, the immunotherapeutic strategy of targeting multiple immune checkpoints is a great challenge to solve based on the genetic and immune status of each patient. In this work [26], they perform immune profiling for two main groups of PM samples for therapeutic decisions: “exclusive” and “depleted” T-cell subtypes (with high levels of PDL-1, TIM-3, galectin-9). An increase in the expression of immune checkpoint molecules has also been reported after neoadjuvant chemotherapy by modifying the microenvironment, this being a beneficial prognostic factor for overall survival [27]. These data provide the possibility of applying chemotherapy combined with immunotherapy or even dual checkpoints.

The latest advances in gastric cancer are aimed at molecular classification. The two most advanced classifications are The Cancer Genome Atlas (TCGA) [28] and the Asian Cancer Research Group [29], which are able to perform prognostic classification, at risk of relapse, such as peritoneal metastases, in addition to a clinical application, selecting the best treatment strategies. Despite using similar procedures, both classifications are not superimposable, and the subgroups determined in them are not equivalent. Only the subtype with microsatellite instability appears to be equivalent in terms of survival in both classifications. The Cancer Genome Atlas (TCGA) classifies GC into four main molecular subtypes: Epstein-Barr virus positive tumors, showing recurrent PIK3CA mutations; extreme DNA hypermethylation and amplification of JAK2; CD274 (PD-L1); and PDCD1LG2 (PD-L2), unstable microsatellite tumors showing high mutation rates genomically stable tumors, which are enriched for diffuse histological variant and RHOA mutations or fusions involving RHO family GTPase activating proteins; and tumors with chromosome instability, showing marked aneuploidy and focal amplification of receptor tyrosine kinases. However, these classifications have not been shown to be useful in clinical practice, and to date only three molecular biomarkers have been shown to predict a response to targeted therapies in GC patients: HER2 positivity for trastuzumab and trastuzumab deruxtecan and MSI and PD-L1 expression for the immune checkpoint. Therefore, for future clinical trials with immunotherapy, we must select immunosensitive patients [30].

## 2. Current Practice

In recent years there has been a transcendental change in the treatment of advanced solid tumours due to immunotherapy, but not for most patients with GC, since the benefit of anti-PD-1 monotherapy is modest and most GC are not immunologically “hot”. The clinical trials include advanced GC, with a 25–30% sample representation with peritoneal carcinomatous. (Table 1)

Following the success of ATTRACTION-2 in 2017 [31], immune checkpoint inhibitors’ efficacy with nivolumab in chemo-refractory gastric cancer compared to best supportive care was demonstrated. However, several negative trials testing immune checkpoint inhibitors have been reported, including KEYNOTE-061 [32] (second line pembrolizumab vs. paclitaxel), JAVELIN-300 [33] (third line avelumab vs. chemotherapy), and JAVELIN-100 [34] (first line avelumab maintenance). The biomarker results from these negative assays demonstrated subsets of patients that were more sensitive to anti-PD-1, including those with high PD-L1 expression, unstable microsatellite tumours, and tumours with a high tumour mutation load.

In the first-line setting, the KEYNOTE-062 [35] trial showed non-inferiority of pembrolizumab compared to chemotherapy in PD-L1 CPS (pooled ratio score) ≥1 patients, but low response rates and detrimental survival early in progression-free pembrolizumab-treated patients. In KEYNOTE-062, pembrolizumab also failed to improve overall survival when added to chemotherapy in PD-L1 CPS ≥1 and ≥10 groups. However, in the first-line treatment, the CheckMate-649 [36] trials showed an overall survival benefit of >3 months for GC patients with PD-L1 CPS ≥5 treated with a combination of oxaliplatin-fluoropyrimidine chemotherapy and nivolumab, and its clinical practice is likely to change. ATTRACTION-4 [37], an Asian study, took a similar approach in a population of all types and, although a progression-free survival benefit was evident, no overall survival benefit was demonstrated. This could be due to a lack of selection of biomarkers or high levels of second-line treatment in Asian patients. Finally, the KEYNOTE-059 [38] trial evaluated cisplatin-based chemotherapy plus pembrolizumab in the first-line, and this demonstrated a survival benefit with the addition of pembrolizumab to chemotherapy, although the benefit for adenocarcinoma is not yet clear. In addition, real-world effectiveness of nivolumab (DELIVER trial) demonstrated a comparable survival time and shows that the presence of peritoneal metastasis was a prognostic factor for OS and PFS [39]. Regarding cytoreductive surgery with hypertermic intraperitoneal chemotherapy (CRS-HIPEC) in gastric peritoneal metastases, the benefit remains unclear [7,40]. The evidence is very controversial, among other things due to the design of the studies. It is important to bear in mind that in 2018 a consensus about CRS-HIPEC in PM was published in which the authors agreed that for GC a prospective randomized trial is needed and that patients with PM from GC should be considered for clinical trials [41,42].

There are several case reports and studies showing the benefit of multimodality therapy including immunotherapy [43,44]. Therefore, multidisciplinary treatment of unresectable gastric cancer is believed to be essential to improve patient outcomes.

## 3. Ongoing Clinical Trials

With next-generation sequencing (NGS) and better genetic profiling, it may be feasible to personalize the systemic therapy regimen; however, currently responses are poor, and the recurrence rates in the peritoneal cavity are still high. Hence, considering the “immunocompetence” of the peritoneum and the oncologic outcomes achieved with checkpoint inhibitors in advanced gastric cancer [45], the need of a research focus on intraabdominal administration of immunotherapeutic drugs is evident.

Limited data has been published with respect to immunotherapy for peritoneal metastasis from gastric cancer, and most of the studies are currently ongoing (Table 2).

The only phase II randomized clinical trial published wanted to address the potential benefit of catumaxomab, a bi-specific (anti-EpCAM, anti-CD3) agent, as a postoperative intraperitoneal immunotherapy [46]. The study had to be stopped prematurely because of toxicity, as one patient died postoperatively of multiorgan failure, and grade 3–5 complications occurred in all of the patients. There are, however, encouraging results as in terms of overall survival.

Lian Lu et al. recently confirmed the efficacy of a combination of camrelizumab, an IgG4κ humanized monoclonal antibody (mAb), anti-angiogenics, and chemotherapy for neoadjuvant/conversion treatment of cT4a/bN + gastric cancer [47]. Patients received camrelizumab (200 mg d1), apatinib (250 mg d1–14), S-1 (50 mg bid d1–10) ± oxaliplatin (85 mg/m^2^) for at least two cycles, followed by re-evaluation and operation. At a median follow-up of 12.5 months (3.4–19.5) 13 of 17 patients (76.5%) with R0 resection were recurrence-free. Based on those results, two randomized controlled phase II trials have been planned, one of them active but not recruiting, to assess the effectiveness and safety of HIPEC, the anti-PD1 antibody Camrelizumab (SHR-1210), an intravenous chemotherapy combined with surgery for conversion therapy in patients with advanced gastric cancer with peritoneal metastasis (NCT04889768). The other one is under active recruitment for the treatment of unresectable metastatic GC (NCT0469418).

Although not recruiting yet, Sorrento Therapeutics opened a phase I clinical trial (NCT03682744) of anti-CEA intraperitoneal CAR-T infusions for treatment in patients with CEA-expressing adenocarcinoma peritoneal metastases or malignant ascites. T cells are activated and then re-engineered to express chimeric antigen receptors (CARs) specific for CEA. Currently, the INSIGHT platform trial has shown to be safe and efficacious for intralesional/peritoneal IMP321 (LAG-3Ig fusion protein and eftilagimod alpha) and avelumab in advanced stage solid tumor entities [48].

## 4. Conclusions and Future Perspectives

Currently, the efficacy of CRS-HIPEC in PM still remains unclear. Following the Chicago Group Consensus in 2018, two randomized phase III trials comparing CRS/HIPEC with CRS alone (CYTO-CHIP and GASTRIPEC-I trials) were published showing contrasting data [40,49]. These controversial results mean the scientific community must keep designing new clinical trials to find out which patients may benefit from this strategy.

Furthermore, we need to precisely analyze the outcomes from new target therapies and immunotherapy to elucidate the futility or not of such multimodal approaches. At the moment, with the approval of nivolumab for second/third line due to the positive results in the 2017 ATTRACTION-2 trial [31], and the recent favourable outcomes in the CheckMate 649 trial placing nivolumab plus chemotherapy as the new standard first line treatment [36], it is crucial to recognize molecular conditions such as TMB, PDL1, MSI and EBV in order to stratify real responders. Even though the immunocompetence of the peritoneum is known, the presence of peritoneal metastases appear as a negative independent prognostic factor in real-world data [39]. Therefore, multiple efforts have to be made to achieve valuable immune control of the peritoneum. Hopefully, results from early phase trials with peritoneal camrelizumab (IgG4) and engineering CART cells will answer some of the key points.

## Figures and Tables

**Table 1:** Summary of clinical trials with immunotherapy in advanced gastric or gastro-oesophageal junction cancer.

CLINICAL TRIAL	Conditions	Molecular Condition	Peritoneum Metastases	Interventions	Results
ATTRACTION-2, phase 3 trial Yoon-Koo Kang (2017) [31]	3rd and successive lines	regardless of PD-L1	19–26%	3 mg/kg nivolumab or placebo IV every 2 weeks	Approved 2°–3° line some countries
KEYNOTE-061, phase 3 trial Kohei Shitara (2018) [32]	2nd line	PD-L1 CPS ≥ 1	28%	pembrolizumab 200 mg every 3 weeks for up to 2 years or standard-dose paclitaxel.	Negative trial
JAVELIN-300, phase 3 trial Y-J Bang (2018) [33]	3rd line	regardless of PD-L1	not specified	avelumab 10 mg/kg IV every 2 weeks or physician’s choice of chemotherapy (paclitaxel 80 mg/m^2^ on days 1, 8, and 15 or irinotecan 150 mg/m^2^ on days 1 and 15, each of a 4-week treatment cycle)	Negative trial
JAVELIN Gastric 100, phase 3 trial Markus Moehler (2021) [34]	1st line avelumab maintenance	PD-L1 CPS ≥ 1	not specified	Patients without progressive disease after 12 weeks of first-line FOLFOX were assigned to avelumab 10 mg/kg every 2 weeks or continued chemotherapy	Negative trial
KEYNOTE-062, phase 3 trial Shitara (2020) [35]	First line	PD-L1 CPS ≥ 1	not specified	pembrolizumab 200 mg, pembrolizumab + chemotherapy (cisplatin 80 mg/m^2^/d on day 1 plus fluorouracil 800 mg/m^2^/d on days 1 to 5 or capecitabine 1000 mg/m^2^ twice daily), or chemotherapy + placebo, every 3 weeks.	Pembrolizumab was noninferior to chemotherapy, but was not superior to chemotherapy for the OS and PFS
CheckMate 649, phase 3 trial Janjigian (2021) [36]	First line	regardless of PD-L1	not specified	nivolumab (360 mg every 3 weeks or 240 mg every 2 weeks) plus chemotherapy (capecitabine and oxaliplatin every 3 weeks or FOLFOX every 2 weeks), nivolumab plus ipilimumab, or chemotherapy alone	Nivolumab is the first PD-1 inhibitor to show superior OS and PFS benefit and an acceptable safety profile, in combination with chemotherapy. Represents a new standard first-line treatment.
ATTRACTION-4, phase 2 trial Boku (2019) [37]	First line	regardless of PD-L1	Not specified	nivolumab (360 mg intravenously every 3 weeks) plus SOX every 3 weeks or CapeOX every 3 weeks	PFS benefit was apparent, no OS benefit was demonstrated. ATTRACTION-4 has proceeded to part 2 (phase III)
KEYNOTE-059, phase 2 trial Bang (2019) [38]	Firstline	PD-L1 CPS ≥ 1	Not specified	Cohort 3(pembrolizumab monotherapy) PDL1 CPS ≥ 1. Cohort 2 (combination therapy) pembrolizumab +. Chemotherapy	OS benefit with the addition of pembrolizumab to chemotherapy, although the benefit for adenocarcinoma is not yet clear.

IV: Intravenously; FOLFOX: oxaliplatin plus a fluoropyrimidine; SOX (S-1orally; oxaliplatin IV); CapeOX (capecitabineorally; oxaliplatin IV); PD-L1: programmed cell death ligand 1; CPS: combined positive score.

**Table 2:** Ongoing clinical trials with immunotherapy in GC.

Rank	NCT Number	Title	Conditions	Interventions	Phases	Completion Date
1	NCT04889768	HIPEC Combined with Camrelizumab, Paclitaxel and S-1 for Conversion Therapy in Patients With Advanced Gastric Cancer With Peritoneal Metastasis	Gastric Cancer, HIPEC, Anti-PD-1 Antibody Camrelizumab (SHR-1210), Chemotherapy and Surgery	Drug: HIPEC, anti-PD-1 antibody Camrelizumab (SHR-1210), Chemotherapy and Surgery	Not Applicable	July 31, 2025
2	NCT04694183	The Conversion Therapy of Chemotherapy Plus Camrelizumab in Metastatic Gastric Cancer	Gastric Cancer	Drug: Paclitaxel + S-1 + anti-PD-1 antibody (Peritoneal metastasis) |Drug: SOX regimen + anti-PD-1 antibody (Liver metastasis, para-aortic lymph node metastasis)	Phase 2	November 17, 2022
3	NCT03682744	CAR-T Intraperitoneal Infusions for CEA-Expressing Adenocarcinoma Peritoneal Metastases or Malignant Ascites (IPC)	Peritoneal Carcinomatosis|Peritoneal Metastases|Colorectal Cancer|Gastric Cancer|Breast Cancer|Pancreas Cancer|Carcinoembryonic Antigen	Biological: anti-CEA CAR-T cells	Phase 1	March 2021
4	NCT03252938	Feasibility and Safety of IMP321 for Advanced Stage Solid Tumors	Solid Tumors|Peritoneal Carcinomatosis	Drug: IMP321|Drug: Avelumab	Phase 1	June 30, 2024
5	NCT01784900	Treatment of Gastric Peritoneal Carcinomatosis by Association of Complete Surgical Resection of the Lesions and Intraperitoneal Immunotherapy Using Catumaxomab	Patients With Gastric Peritoneal Carcinomatosis	Drug: Catumaxomab 100 µg|Drug: Catumaxomab 140 µg	Phase 2	January 2016

## Data Availability

The study did not report any data.
